# Scoring model to predict postoperative neurological deterioration in spinal schwannoma

**DOI:** 10.3389/fonc.2023.1086299

**Published:** 2023-03-14

**Authors:** Zongchi Liu, Zihan Xu, Jie Shen, Tiesong Zhang, Hongwei Lin, Lihui Zhou, Fan Wu, Luyuan Zhang, Yuxiang Weng, Renya Zhan, Yu Zhu, Jiangbiao Gong

**Affiliations:** ^1^ Department of Neurosurgery, First Affiliated Hospital, College of Medicine, Zhejiang University, Hangzhou, China; ^2^ Department of Neurosurgery, HwaMei Hospital, University of Chinese Academy of Sciences, Ningbo, China

**Keywords:** spinal schwannoma, spinal surgery, functional outcome, scoring model, prognosis

## Abstract

**Background:**

Spinal schwannomas (SSs) are benign tumors affecting the nerve sheath, accounting for 25% of spinal nerve root tumors. Surgery represents the mainstay of treatment for SS patients. Following surgery, approximately 30% of patients experienced developed new or worsening neurological deterioration, which probably represented an inevitable complication of nerve sheath tumor surgery. The objective of this study was to identify the rates of new or worsening neurological deterioration in our center and accurately predict the neurological outcomes of patients with SS by developing a new scoring model.

**Methods:**

A total of 203 patients were retrospectively enrolled at our center. Risk factors associated with postoperative neurological deterioration were identified by multivariate logistic regression analysis. β–coefficients for independent risk factors were used to define a numerical score to generate a scoring model. The validation cohort at our center was used to verify the accuracy and reliability of the scoring model. Receiver operating characteristic (ROC) curve analysis was used to evaluate the performance of the scoring model.

**Results:**

In this study, five measured variables were selected for the scoring model: duration of preoperative symptoms (1 point), radiating pain (2 points), tumor size (2 points), tumor site (1 point), and dumbbell tumor (1 point). The scoring model divided the spinal schwannoma patients into three categories: low risk (0-2 points), intermediate risk (3-5 points), and high risk (6-7 points), with predicted risks of neurological deterioration of 8.7%, 36%, and 87.5%, respectively. And the validation cohort confirmed the model with the predicted risks of 8.6%, 46.4%, and 66.6%, respectively.

**Conclusion:**

The new scoring model might intuitively and individually predict the risk of neurological deterioration and may aid individualized treatment decision-making for SS patients.

## Introduction

Spinal schwannomas(SSs) originating from spinal nerve root sheaths are the most common benign tumors that occur in the spinal intradural and extramedullary space, comprising 25% of spinal nerve root tumors (1), and with an annual incidence of around 0.3−0.5 cases/100,000 individuals (2). Morphologically, spinal schwannomas are usually enveloped, round or nearly round, with a distinctive dumbbell pattern if squeezed out of the spinal canal (3). Advances in neuroimaging, especially MRI, have led to an increasing number of incidental detections of SSs. In general, most tumors except intratumoral hemorrhage show low signal on T1-weighted images and high signal on T2-weighted images (4, 5). SS on T1-weighted Gd-enhanced MR images could be detected as homogenous, heterogeneous and rim enhancement (4). Usually, SS is slow growing that causes mild symptoms in early stages. As tumors grow, oppression to nerve roots or spinal cord can cause pain and neurological deficits. The common initial symptoms include local pain (usually back pain) and radiating pain that can involve limbs (6). Manifestations of neurological deficits include sensory (weakness and numbness) and motor findings (weakness, fasciculations and atrophy) (7).

Total surgical resection is the standard treatment for SS (8). Yet due to the anatomical limitations of surgical operation in the spinal canal and adhesion of the tumor to nerve roots and spinal cord, more than 20% patients after surgery suffered postoperative neurological deterioration (9). Although some of these newly-developed or worsened deficits partially alleviate within several months, high incidence of operation-related neurologic injuries and functional impairments persist for years and remain an unsolved problem that brings significant challenges for surgeons (10). Few studies have indicated several risk factors for poor prognosis after spinal schwannoma resection, yet the factors analyzed in the published literature were quite limited, and currently there is lack of precise models and practical tools to predict personalized risk for postoperative neurological deterioration.

In this study, we developed and internally validated a novel scoring model for prediction of postoperative neurological deterioration based on preoperative information in a retrospective cohort of spinal schwannoma patients. This scoring model may be useful for clinical decision-making in patients with spinal schwannoma and for risk stratification of patients associated with postoperative neurological deterioration in future.

## Materials and methods

### Study design and population

**Figure 1:**
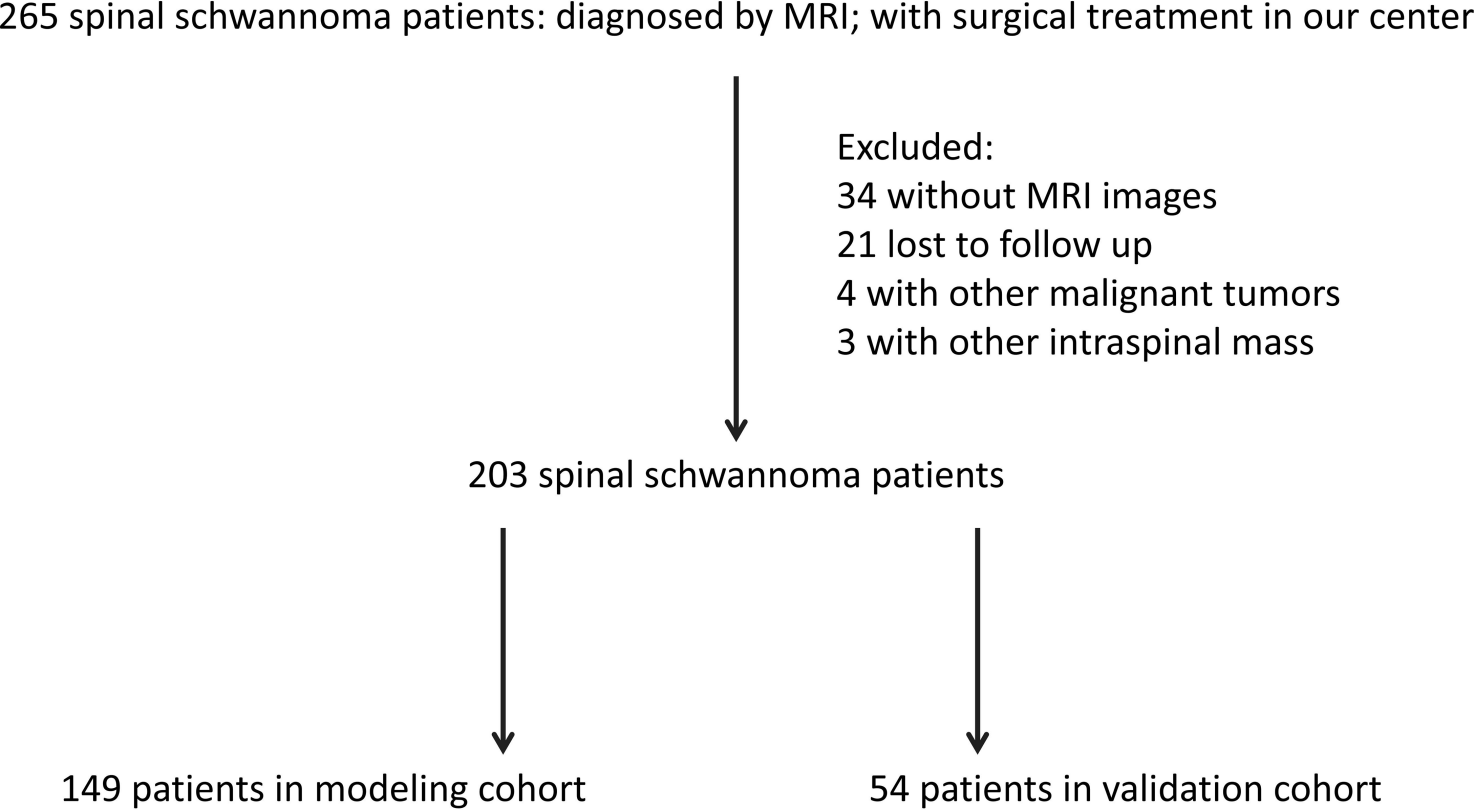
Flow diagram of the included patients.

Between January 2013 and December 2021, a total of 265 patients with non-syndromic spinal schwannoma were comprised in our study, who were treated in the Department of Neurosurgery at our center. The inclusion criteria were: (1) spinal schwannoma was confirmed by pathological diagnosis after surgery; (2) all patients received and signed the informed consent; (3) clinical data and follow-up data were complete. The exclusion criteria were: (1) patients with other intraspinal tumors; (2) patients with other types of malignancies, chronic diseases, and other bone diseases; (3) patients who underwent surgical treatment in referral centers; (4) patients lost during follow up or with incomplete data; (5) patients with neurofibromatosis [diagnosed by related diagnostic criteria (11)]. According to the inclusion and exclusion criteria, 203 patients were included in the final analysis (**Figure 1**). The patients were randomly divided into a modeling cohort (n = 149) and a validation cohort (n = 54). After spinal schwannoma surgery, clinical follow-up was performed at 12 months. This study was approved by the ethics committee of First Affiliated Hospital of Zhejiang University (11T20220373B).

### Evaluation

We collected all the clinical data of patients through the electronic medical record system. Detailed clinical information of every patient was recorded, including patient age, gender, preoperative neurologic deficits, duration of symptoms, and neurologic improvements. The preoperative neurologic deficits included back pain, radiating pain, motor weakness, sensory disturbance of the limbs, gait disturbance, and urinary disturbance. Moreover, the degrees of preoperative clinical symptoms were evaluated by the McCormick score. Imaging features were also collected, including tumor size (the maximum tumor diameter in cranial-caudal direction), tumor number, tumor level, number of vertebral segments involved, cystic change, and enhancement. Images were analyzed and reported separately by two different doctors and any conflicting items were reviewed and given the final decision by the third senior doctor.

### Outcome measures

A follow-up evaluation was performed 12 months after the operation *via* telephone and/or outpatient appointment. We collected the postoperative neurological outcome and evaluated tumor recurrence based on MRI and/or contrast-enhanced MRI. Presence of neurological deterioration was defined as any newly developed or worsening preoperative abnormality in patients’ sensory or motor function after surgery. Tumor recurrence was defined as tumor reappearance on MRI during follow-up after confirmation of tumor gross total resection on initial postoperative MRI.

### Statistical analysis

Data were analyzed using the SPSS Version 23.0 software (IBM Company, Armonk, NY, USA). Continuous variables were reported as mean ± standard deviation and compared between groups using an unpaired t-test. Categorical variables were presented as percentages and the chi-square test was used to compare categorical versus categorical variables. Univariate and multivariate logistic regression analyses were performed using neurological deterioration and recurrence as the outcome variable in the derivation cohort. All variables with p-value ≤ 0.1 in the univariate analysis were entered into the multivariate logistic regression analysis to identify variables independently associated with neurological deficits. Risk variables independently associated with prognosis were entered into the new scoring model. The points assigned to the variables in the scoring model were assigned based on the beta coefficients from the logistic regression models. Beta coefficient was regarded as the coefficient of different classification levels of individual factors in the model. Finally, the patient’s disease risk was evaluated by calculating the total score. The discrimination of the scoring model was assessed by the area under the receiver operating characteristic curve (AUC) and the Hosmer-Lemeshow test was used to evaluate the model fit (Hosmer-Lemeshow statistic ≥ 0.05).

## Results

### Basic information of patients

**Figure 2:**
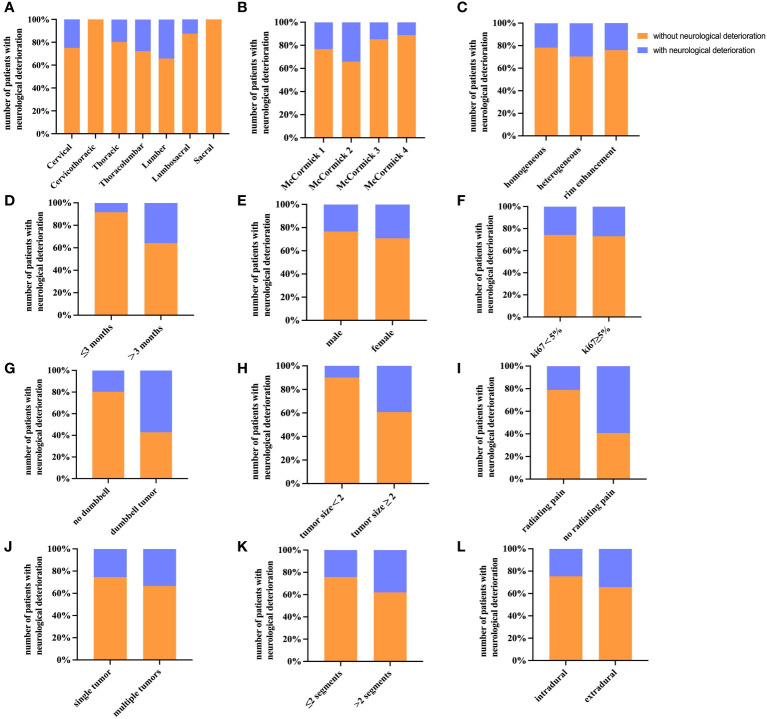
Histograms of different factors for patients with postoperative neurological deterioration. The histograms show the number of patients with postoperative neurological deterioration and their percentage. **(A)** Tumor site (sagittal topography). **(B)** Preoperative McCormick score. **(C)** Contrast-enhanced MRI. **(D)** Duration of symptoms. **(E)** Gender. **(F)** Ki67 labeling index. **(G)** Dumbbell tumor. **(H)** Tumor size. **(I)** Preoperative radiating pain. **(J)** Multiple tumors. **(K)** Number of involved segments. **(L)** Tumor location.

The overall study population included a total of 203 patients, with 107 men and 96 women. The average age at evaluation for this study was 52.6 years (SD=14.2). Local pain (79.8%) is the most common complaint of patients seeking neurosurgeon consultations. Other common complaints include paresthesia (37.9%), motor deficit (23.6%), and radiating pain (13.3%). The mean duration of preoperative symptoms for all patients enrolled was 10.6 months (SD=15.7). In this study, spinal schwannoma was most common in lumber (39.4%), followed by thoracic (26.1%) and cervical (17.7%). 53 patients (26.1%) developed neurological deterioration, and 17(8.3%) developed local recurrence within the follow-up period. The mean follow-up point for first postoperative neurological deterioration was 32.22 ± 14.78 days. All relevant demographic and clinical signs of the study participants are shown in **Table 1**. In addition, the distribution of other factors among the 203 spinal schwannoma patients with postoperative neurological deficits is shown in **Figure 2**. Notably, postoperative neurological deterioration appeared to be associated with patients who suffered radiating pain, larger tumors, and longer duration of preoperative symptoms.

**Table 1:** Baseline data and clinical presentation. .

	n	%
Total no. of patients	203	100%
Sex		
Male	107	52.7%
Female	96	47.2%
Age(years)	52.4 ± 14.2	
Preoperative neurological deficits		
Local pain	162	79.8%
Radiating pain	27	13.3%
Motor deficit	48	23.6%
Paresthesia	77	37.9%
Sphincter impairment at first evaluation		
Yes	6	2.9%
No	197	97%
Urinary retention		
Yes	3	1.4%
No	200	98.5%
Mean duration of preoperative symptoms(months)	10.6 ± 15.7	
Sagittal topography		
Cervical	36	17.7%
Cervicothoracic junction	4	2.0%%
Thoracic	53	26.1%
Thoracolumbar junction	18	8.9%
Lumber	80	39.4%
Lumbosacral junction	8	3.9%
Sacral	4	2.0%
Spinal localization		
Intradural	171	84.2%
Extradural	32	15.8%
Dumbbell shape	35	17.2%
Tumor characteristic (Contrast enhanced MR imaging)		
Homogeneous	55	27.1%
Heterogeneous	98	48.3%
Rim enhancement	50	24.6%
Neurological deterioration	53	26.1%
Postoperative recurrence	17	8.4%

### Tumor recurrence

**Figure 3:**
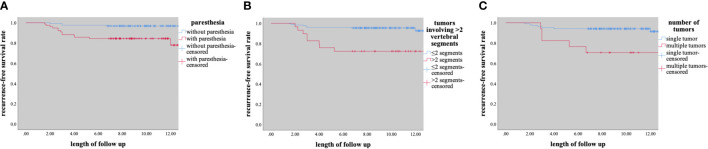
Kaplane-Meier curves of recurrence for **(A)** paresthesia, **(B)** tumors involving ≥2 segments, **(C)** number of tumors.

Univariate analysis showed that preoperative paresthesia, multiple tumors, and tumors involving > 2 vertebral segments were significantly associated with tumor recurrence (paresthesia & multiple tumors, p<0.001; tumors involving > 2 vertebral segments, p=0.001; **Table 2**). As is shown in **Table 2**, multivariate analysis was performed by using a binary logistic regression. Results showed that paresthesia and multiple tumors were independent risk factors for tumor recurrence. Multiple tumors, however, showed borderline significance for the tumor recurrence in multivariate analysis. Surprisingly, tumor size was not a significant risk factor for tumor recurrence (p = 0.752) (**Table 2**). The Kaplane-Meier curves of tumor recurrence for paresthesia, tumors involving > 2 vertebral segments, and multiple tumors were shown in **Figure 3**.

**Table 2:** Univariate and Multivariate logistic regression model for tumor-recurrence.

		Univariate analysis		Multivariate analysis	
Factors	N	% or Median	P value	OR	P value
Age, <50/≥50	82/121	60.2% vs 52.9%	0.559		
Sex, male/female	107/96	45.7% vs 64.7%	0.133		
Preoperative neurological deficits					
Local pain	41/162	80.1% vs 76.5%	0.721		
Radiating pain	176/27	14.5% vs 0%	0.092		
Motor deficit	155/48	23.7% vs 23.5%	0.991		
Paresthesia	126/77	34.4% vs 76.5%	**0.001**	7.006	**0.002**
Duration of symptoms,≤3/>3 months	72/131	64.5% vs 64.7%	0.988		
Tumor size, ≤2/>2	91/112	54.8% vs 58.8%	0.752		
Mono/multiple tumors	185/18	6.5% vs 35.3%	**<0.001**	4.319	**0.044**
Cystic degeneration, yes/no	16/186	8.6% vs 0%	0.206		
No. of involved segments, ≤2/>2 segments	174/29	11.3% vs 47.1%	**<0.001**	4.862	**0.015**
Preoperative McCormick score, (1-2)/(3-4)	160/43	20.4% vs 29.4%	0.386		
Contrast-enhanced MRI			0.659		
Homogeneous	55/203	26.3% vs 35.3%			
Heterogeneous	98/203	48.4% vs 47.1%			
Rim enhancement	50/203	25.3% vs 17.6%			
Dumbbell tumor, yes/no	35/168	17.7% vs 11.8%	0.532		
Location, intradural/extradural	171/32	16.1% vs 11.8%	0.636		
Ki67 labeling index,<5%/≥5%	121/82	40.9% vs 35.3%	0.654		

The bold values indicate that the factors are statistically significant.

### Postoperative neurological deterioration

In total, 149 patients were included in the modeling cohort and 54 patients were included in the validation cohort. As shown in **Table 3**, the demographics of the modeling group indicated that 79(53.02%) were male, 70(46.98%) were female and the mean age was 53 years (range 22-68 years). At 12-month follow-up, 77.35% patients with postoperative neurological deterioration had improved, and 24.53% patients had recovered completely. **Table 3** showed that in univariate analysis, postoperative neurological deterioration was associated with duration of preoperative symptoms >3 months (P<0.001), radiating pain (P<0.001), preoperative McCormick score (P=0.027), tumor dumbbell shape (P<0.001) and tumor size >2cm (P<0.001) in the modeling cohort. Variables with a P value of <0.1 in univariate analysis from the modeling group were subsequently entered into the multivariate logistic regression model (**Table 4**). Results showed that duration of preoperative symptoms (OR, 4.013; P=0.026), radiating pain (OR, 4.716; P=0.010), sagittal topography (OR, 2.841; P=0.035), dumbbell shape (OR, 3.834; P=0.016) and tumor size (OR, 4.876; P=0.004) were independent risk factors for postoperative neurological deficits. The Hosmer-Lemeshow test indicated a good fit of the model (P=0.295, **Table 4**).

**Table 3:** Demographic and baseline characteristics of the study population and univariate analysis results of modeling cohorts and validation cohorts.

Variable	Modeling cohort		P value	Validation cohort		P value
	With deterioration	Without deterioration		With deterioration	Without deterioration	
Age	52.69 ± 15.10	52.46 ± 13.84	0.931	54.29 ± 13.60	50.89 ± 14.86	0.426
Gender			0.423			0.633
Male	17 (47.2%)	62 (54.9%)		8 (47.1%)	20 (54.1%)	
Female	19 (52.8%)	51 (45.1%)		9 (52.9%)	17 (45.9%)	
Duration of preoperative symptoms			**<0.001**			**0.023**
<3 months	4 (11.1%)	50 (44.2%)		2 (11.8%)	16 (43.2%)	
>3 months	32 (88.9%)	63 (55.8%)		15 (88.2%)	21 (56.8%)	
Preoperative neurological deficits						
Local pain	28 (77.8%)	88 (77.9%)	0.990	15 (88.2%)	31 (83.8%)	0.669
Radiating pain	11 (30.6%)	7 (6.2%)	**<0.001**	5 (29.4%)	4 (10.4%)	0.088
Motor deficit	9 (25%)	27 (23.9%)	0.893	4 (23.5%)	8 (21.6%)	0.876
Paresthesia	12 (33.3%)	43 (38.1%)	0.609	6 (35.3%)	16 (43.2%)	0.581
Sphincter impairment	1 (2.8%)	4 (3.5%)	0.825	0	1 (2.7%)	0.494
Urinary retention	0	3 (2.7%)	0.323	0	0	/
McCormick score			**0.027**			0.736
1-2	3 (8.3%)	29 (25.7%)		14 (82.4%)	29 (78.4%)	
3-4	33 (91.7%)	84 (74.3%)		3 (17.6%)	8 (21.6%)	
Sagittal topography			0.050			0.216
Lumber	19 (52.8%)	39 (34.5%)		9 (52.9%)	13 (35.1%)	
Other segments	17 (47.2%)	74 (65.5%)		8 (47.1%)	24 (64.9%)	
Spinal localization			0.170			0.917
Intradural	27 (75%)	96 (85%)		15 (88.2%)	33 (89.2%)	
Extradural	9 (25%)	17 (15%)		2 (11.8%)	4 (10.8%)	
Dumbbell shape	14 (38.9%)	11 (9.7%)	**<0.001**	6 (35.3%)	4 (10.8%)	**0.031**
Tumor characteristic (Contrast enhanced MR imaging)			0.937			0.081
Homogeneous	10 (27.8%)	33 (29.2%)		2 (11.8%)	10 (27%)	
Heterogeneous	17 (47.2%)	55 (48.7%)		12 (70.6%)	14 (37.8%)	
Rim enhancement	9 (25%)	25 (22.1%)		3 (17.6%)	13 (35.1%)	
No. of involved/>2 segments			0.938			0.041
≤2 segments	13 (36.1%)	40 (35.3%)		12 (70.6%)	34 (91.9%)	
>2 segments	23 (63.8%)	73 (64.6%)		5 (29.4%)	3 (8.1%)	
Multiple tumors	3 (8.3%)	8 (7.1%)	0.802	3 (17.6%)	4 (10.8%)	0.487
Tumor size			**<0.001**			**0.012**
≤2	6 (16.7%)	62 (54.9%)		3 (17.6%)	20 (54.1%)	
>2	30 (83.3%)	51 (45.1%)		14 (82.4%)	17 (45.9%)	
Cystic degeneration	1 (2.8%)	7 (6.2%)	0.428	2 (11.8%)	6 (16.2%)	
Ki67 index			0.699			0.743
<5%	21 (58.3%)	70 (61.9%)		10 (58.8%)	20 (54.1%)	
≥5%	15 (41.7%)	43 (38.1%)		7 (41.2%)	17 (45.9%)	
Somatosensory and motor evoked potentials	35 (97.2%)	105 (92.9%)	0.363	15 (88.2%)	36 (97.2%)	0.214

The bold values indicate that the factors are statistically significant.

**Table 4:** Multivariate logistic regression model for post-operative neurological deterioration.

Variable included in model	S.E	OR	95%CI	P
Duration of preoperative symptoms	0.623	4.013	1.183-13.614	0.026
Radiating pain	0.604	4.716	1.444-15.403	0.010
Sagittal topography	0.497	2.841	1.073-7.519	0.035
Dumbbell shape	0.558	3.834	1.286-11.437	0.016
Tumor size	0.557	4.876	1.636-14.536	0.004
X²				9.596
Degree of freedom				8
*P*				0.295

Of the 54 patients in the validation cohort, 17 (31.5%) patients had postoperative neurological deterioration after the 12-month follow-up. Detailed data of the validation cohort are shown in **Table 3**.

Due to the higher risk of nerve injury in C5 schwannomas/plexus-associated schwannomas (C5-T1) compared to other cervical schwannomas (12). In our cohort, there were 2.5% at C5 and 9.9% at C5-T1. We performed univariate analyses for C1-C4 and C5-T1 (P=0.073).

### Development of the scoring model

A scoring system designated Spinal-Schwannoma Postoperative Neurological Deterioration Scoring System (SPNDSS) was constructed to predict postoperative neurological deterioration based on multivariate analysis results of the modeling cohort (**Table 5**). The total scores could be calculated by the sum of the following 5 variables: total score = (duration of preoperative symptom more than 3 months: 1) + (preoperative radiating pain: 2) + (tumor size larger than 2cm: 2) + (tumor occur in Lumbar spine: 1) + (Dumbbell shape: 1). In this scoring system, a scale ranging from 0 to 7 based on the scores assigned from the β-coefficient of each variable was designed. The total score of the new scoring system can be divided into three different categories as outlined as follows (**Table 6**): Low risk stratification (0-2 point; n=80), 8.7% prediction risk of postoperative neurological deterioration; moderate risk stratification (3–5 point; n=61), 36% prediction risk of postoperative neurological deterioration in patients; high risk stratification (6–7 point; n=8), 87.5% prediction risk of postoperative neurological deterioration in patients.

**Table 5:** Scoring System derived from the β coefficients.

Variable included in model	Categories	β coefficient	Score
Duration of preoperative symptoms	≤3 month >3 month	0 (reference) 1.389	0 1
Radiating pain	No radiating pain with radiating pain	0 (reference)1.551	0 2
Tumor size	<2 cm ≥2 cm	0 (reference) 1.584	0 2
Tumor site	Other spine Lumbar spine	0 (reference) 1.044	0 1
Dumbbell tumor	No dumbbell Dumbbell	0 (reference) 1.344	0 1

**Table 6:** Risk of neurological deterioration for low, moderate, and high-risk individuals according to the score model.

Table grid	Score	Observed risk (validation cohort)	Predicted risk	OR (95% CI)
Low risk stratification	0-2	8.6%	8.7%	1 (reference)
Moderate risk stratification	3-5	46.4%	36%	5.214 (1.977-13.751)
High risk stratification	6-7	66.6%	87.5%	20.857 (5.977-72.777)

### Discrimination and calibration of the scoring system

**Figure 4:**
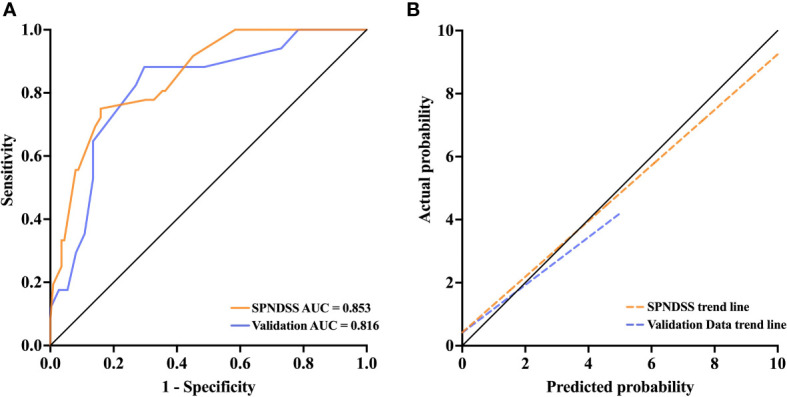
**(A)** The AUC of the SPNDSS was 0.853 (95% CI: 0.786–0.919), while it was 0.816 (95% CI: 0.693–0.939) in validation data. **(B)** The SPNDSS had a good calibration in derivation cohort and validation cohort.

According to the SPNDSS, of the 54 patients in the validation cohort, 23 patients have been classified as low-risk stratification (score 0–3), 28 patients have been classified as moderate risk stratification (score 3–5) and 3 patients have been classified as high-risk stratification (score 6–7). The prediction risks of all three groups were 8.6%, 46.4% and 66.6%, respectively (**Table 6**). In the modeling cohort, the AUC of the SPNDSS was 0.853 (95% CI: 0.786–0.919; **Figure 4**). The validation cohort also showed good discrimination, with an AUC of 0.816 (95% CI: 0.693–0.939; **Figure 4**). The Hosmer–Lemeshow test showed good calibration in the modeling cohort and validation cohort (P=0.285; P=0.458; **Figure 4**).

## Discussion

Spinal schwannomas are the most common nerve sheath tumors of the spine, arising from Schwann cells (13). The incidence of spinal schwannomas varies with age and the peak incidence is in the fourth and fifth decades of life (14). They have a complex three-dimensional structure as they grow within the spinal canal. Complete surgical resection is the gold standard in the treatment of spinal schwannoma. Despite the high incidence of spinal schwannomas in the spinal canal, little is known about factors that are associated with schwannoma prognosis after surgery. In the current study, we developed and internally validated a new scoring model for the prediction of postoperative neurological deterioration in patients with SS: The SPNDSS scoring model. We believed that this validated scoring model can be used as a practical tool to preoperatively identify risk stratification in patients with SS who need surgical treatment. As far as we know, this was the first study to develop a scoring model to evaluate prognosis for patients with SS.

In our study, 53(26.1%) SS patients got postoperative neurological deterioration and this incidence was largely consistent with those reported by another study(27.7%) (10). In this study we found pain to be the most common symptom of spinal schwannoma and often persists until tumor resection. As early clinical presentation of SS can be nonspecific, thus early local pain can be oftenly misdiagnosed as spinal meningioma, ependymoma, and astrocytoma (7). The pain caused by SS was usually due to compression and impingement of the spinal nerve and/or neighboring neural elements located within or in the vicinity of the spinal canal (15). As tumors gradually progressed, significant spinal cord compression caused paresthesia, motor deficits, and gait disturbances. In the current study, paresthesia was found the second most common symptom. Previous data showed that 70% of spinal schwannoma arose from sensory root, 20% from motor root, and rested from a mixture of both (16). Thus, abnormal motor function was relatively uncommon in the complaints of SS patients and usually occurred when tumors cause severe spinal cord compression. Several studies reported the lumbar spine and thoracic spine as the most frequent sites of SS (17, 18), which was similar to the findings in our study that lumbar spine (39.4%) and thoracic spine (26.1%) were the most common sites of SS. One possible reason was that the length of the nerve roots increased from the high cervical region to the sacral region and it reached the maximum length in the lumbar (19). Sacrum appeared to be a relatively uncommon site for spinal schwannoma occurrence and only accounted for 1.9% of all sites in our study, which was similar to reports of previous study (20).

Our study showed that the recurrence rate of SS was 8.3%, which was within the reported range of 5%-10% in previous studies (21). Fehlings et al. reported that tumor size and tumor involving additional levels were the key predictors of tumor recurrence (15), which had been validated in a recent study (22). In line with these results, our study showed a significant trend of tumor recurrence in patients with tumors involving > 2 vertebral segments. The high recurrence rate may partly attribute to the difficulty of tumor total resection due to the large size of schwannoma at multiple segments. We also found that tumor recurrence was significantly more likely in patients with multiple tumors. As multiple tumors can reflect a status of genetic instability (23) of the patient which may lead to acceleration of residual tumor cells to regrow after surgical resection, it was reasonable that multiple tumors were associated with higher recurrence rate as we observed in this study. Ki67 labeling index detected by immunohistochemistry is currently the most frequently used marker to estimate tumor cell proliferation capability. Li et al. suggested that a cut-off value of >5% for the Ki67 labeling index was an indicator of postoperative recurrence and poor survival (21). Yu et al. reported that more than 60% of patients with a Ki-67 labeling index of >2% exhibit recurrence (24). However, our study showed that a cut-off value of 5% for the Ki67 labeling index was not a risk factor for tumor recurrence at 12 months. We assumed that this may be related to relatively short follow-up time. We analyzed the relationship between tumor recurrence and contrast MRI image features by performing the contrast signal characteristics and found that there was no statistical difference between them.

Based on the existing results, various rates of postoperative neurological deterioration have been demonstrated. Ando et al. noted that the rates of postoperative motor and sensory function deterioration were 13.1% and 20.5% for spinal schwannoma patients, respectively (9). Yamane et al. reported that patients with permanent postoperative neurological deterioration had 35% of patients (12). In our study, the rate of postoperative neurological deterioration of patients in the modeling cohort and validation cohort both exceeded 20%. In our modeling cohort, some significant differences were found: duration of preoperative symptoms, preoperative radiating pain, dumbbell shape, tumor size, and tumor site. And we also found a significant difference in univariate analysis, which disappeared in multivariate analysis: McCormick score. As outlined previously, the compression of the spinal cord by spinal schwannoma was a gradual process. The long duration of preoperative symptoms indicated long-standing spinal cord compression and nerve root damage, so patients had a strong tendency to occur postoperative neurological deterioration after surgical resection. Due to the fact that schwannoma developed more often in sensory nerves rather than motor nerves, preoperative radiating pain may predict the occurrence of postoperative neurological deterioration. For preoperative radiating pain to be a good predictor, we believe it is due to several reasons: 1. Local pain and radiating pain are more caused by intradural tumors. It has been demonstrated that intradural tumors affect the tumor-involved nerve roots and frequently stimulate the surrounding nerve roots (25), and there are significantly more intradural tumors in our cohort (84.2%). Patients with preoperative local and radiating pain were more common in our cohort than patients with preoperative motor dysfunction. Therefore, we think that local and radiation pain should be paid more attention. 2. Motor weakness rarely occur as the first symptom in the lumbosacral region (19). 3. According to our definition of postoperative neurological deterioration, new or deteriorated sensory symptoms are more common than motor dysfunction in our cohort (73.5% vs 20.7%, 5.6% developed both), which is similar to other studies (10, 26). 4. The reason why there is no difference in motor function deterioration may be limited by the number of cases. Dumbbell-shaped tumors usually formed when the tumor progressed to a larger size and protruded out of the intervertebral foramen (27), which posed a challenge for full-cut surgery. It was also demonstrated by Safaee et al. that dumbbell-shaped tumors commonly caused high rates of postoperative sensory function deterioration (26). When dumbbell-shaped spinal schwannomas were removed in surgery, any functional nerve fibers beneath the tumor epineurium were sectioned at the same time, causing postoperative neurologic deterioration (28). For dumbbell tumors, other studies considered that affected nerve roots at the cervical and the lumbar spine should be preserved as there was a high risk of postoperative motor function deterioration and thoracic nerve roots caused only mild postoperative sensory loss after resection (29). We found that tumor occurring in the lumbar spine was an independent risk factor for postoperative neurological deterioration. Possible explanations for this finding were that spinal schwannomas commonly originated from the dorsal sensory roots of the cervical and lumbar spine, and the conventional surgical approach to spinal schwannomas needed to strip paraspinal muscles and cut off nerve roots of tumor origin, which may cause a large trauma range and destroy the normal structure of lumbar spine (8, 27). We think it was very necessary to use intraoperative electrophysiological monitoring during surgical resection of spinal schwannoma. We used intraoperative electrophysiological monitoring in 191 patients, and the utilization rate was 94.09%. For the few patients in whom intraoperative electrophysiological monitoring was not used, it was due to the small size and ease of surgical total resection of the lesion as assessed by the surgeons. We conducted a statistical analysis, but the results showed no significant difference. We think it is due to our high utilization of intraoperative electrophysiological monitoring.

The SPNDSS items included duration of preoperative symptoms, preoperative radiating pain, tumor size, tumor site, and dumbbell-shape tumor were primarily used to assess the risk of postoperative 12-months neurological deterioration. As a tool to assess the risk prediction of postoperative neurological deterioration in patients, SPNDSS had a good predictive ability. Generally, the higher score in the SPNDSS, the more likely the patients will develop neurological deterioration after surgery. To our knowledge, there were no generic scoring models to assess risk prediction of postoperative neurological deterioration. At present, several studies have proposed prediction models for patients with neurological deterioration, but most of these models assessed different risk factors and had limitations in reporting calibration and validation (9, 12, 25). The present study shows that the SPNDSS developed with identified risk factors can very well predict the future risk of postoperative neurological deterioration in spinal schwannoma patients. Furthermore, it can help guide risk patient stratification and management optimization, and may also reduce the cost of multifaceted management of higher-risk patients and lower-risk patients.

Some potential limitations of our study need to be discussed. First, our study was a single-center retrospective observational study. Second, our study only divided the single-center data into a model cohort and a validation cohort for internal verification, lacking external validation. Further studies with more patients and external multicenter data are required for more definite conclusions. With regard to postoperative neurological outcomes, we selected 12 months after surgery as the follow-up point. However, if long-term follow-up date can be acquired, the prediction of postoperative neurological deterioration will be more accurate. In addition, we found relatively few clinical prediction models for spinal schwannoma, which considering further study of this aspect will be the future direction.

## Data availability statement

The raw data supporting the conclusions of this article will be made available by the authors, without undue reservation.

## Ethics statement

The studies involving human participants were reviewed and approved by First Affiliated Hospital, school of Medicine, Zhejiang University. The patients/participants provided their written informed consent to participate in this study.

## Author contributions

ZL and ZX contributed to writing the manuscript. JS and TZ did the data analysis and interpretation of the data. HL, LiZ, and FW contributed to the acquisition of follow-up data. LuZ and YW contributed to the literature review. RZ contributed to the preliminary revision of the manuscript content. YZ and JG contributed to the critical revision of the manuscript for intellectual content. All authors contributed to the article and approved the submitted version.
